# Truth telling and truth witnessing: results from a transformative experiential learning program between Aboriginal Elders and non-Aboriginal researchers

**DOI:** 10.1080/00049530.2024.2425624

**Published:** 2024-11-14

**Authors:** Pat Dudgeon, Angela Ryder, Jemma Collova, Belle Selkirk, Kate Derry, Colin Hansen, Fred Penny, Cheryl Phillips, Marie Pryor, Margaret Taylor, Joanna Alexi, Ee Pin Chang, Craig D’Mello, Shraddha Kashyap, Monique Platell, Helen Milroy

**Affiliations:** a School of Indigenous Studies, The University of Western Australia, Perth, Australia; b Langford Aboriginal Association, Langford, Australia; c Suicide Prevention Australia, Sydney, Australia; d Medical School, The University of Western Australia, Perth, Australia

**Keywords:** Reflexivity, cultural exchange, Aboriginal health and wellbeing, truth-telling, elder knowledges

## Abstract

**Objective:**

Aboriginal Elders have supported Aboriginal health and wellbeing for generations. Aboriginal Elders also play an important role in guiding those who work in health systems to work in culturally safe ways. The Cultural Exchange Program was developed to encourage reflexivity among non-Aboriginal researchers (*N* = 6) through experiential learning and relationship building with local Noongar Elders (*N* = 5). This paper examines the transformative impacts of this program for both the Aboriginal Elders and non-Aboriginal participants.

**Method:**

The Cultural Exchange Program was developed through an Aboriginal Participatory Action Research (APAR) approach. The Elders and researchers had active involvement and influence over the research process and interpretation of data. Themes were co-created through the APAR approach, including a reflexive thematic analysis and feedback loop.

**Results:**

For the Elders, the program promoted a desire to educate and bring about change, facilitated healing through truth-telling, and led to experiences of cultural respect. For the non-Aboriginal participants, the program evoked deep respect for the Elders and their knowledges, surfaced unsettling colonial realities, encouraged an inwards reflection, and motivated social justice change.

**Conclusions:**

These results highlight the transformative and healing experiences which can exist at the intersection of reflexivity, truth-telling, truth-witnessing, and relationship building.

## KEY POINTS

**What is already known about this topic:**

1. The history and practice of health and wellbeing research has contributed to the colonisation of Aboriginal peoples.
2. There is a need for health and wellbeing professionals to develop reflexive skills to work in culturally safe ways with Aboriginal peoples.
3. Aboriginal Elders can play an important role in guiding those who work in health and wellbeing systems to work in culturally safe ways.

**What this topic adds**:

1. This paper outlines the process and outcomes of a Cultural Exchange Program, which encouraged reflexivity among non-Aboriginal mental health and wellbeing researchers.
2. Aboriginal Elders and non-Aboriginal participants engaged in independent but intertwined transformative journeys, through building friendships, trust, and reciprocity.
3. In line with an Aboriginal Participatory Action Approach, Elders and participants co-created themes as co-researchers.

## Introduction

Ngany Kudjal Moorditj, Noonuck Kudjal Moorditj, Ngullang Kudjal Moorditj Yey.

I’m too solid, you’re too solid, we’re too solid now Yey.

Extract from Song in Noongar Language – Fred Penny, Noongar Elder.

### Positionality

We acknowledge the traditional custodians of the Noongar Boodja (Country), where this project occurred. We pay our respect to Elders past and present, the backbone of our communities. We acknowledge their hard work and resilience, and often silent achievements. We also acknowledge the continuing culture, strength and resilience of all Aboriginal and Torres Strait Islander peoples and communities. This paper honours the Noongar Elders who transformed the minds and hearts of the non-Aboriginal researchers in this project.

We recognise our positionality and the ways in which our unique differences shape our perspectives and values. The authors are a collective of senior Aboriginal researchers (H.M., P.D., A.R.), Noongar Elders (C.H., F.P., C.P., M.P., M.T.), and early-to-mid career Aboriginal (B.S) and non-Aboriginal (J.C., K.D., J.A., E.P.C., C.D., S.K., M.P.) researchers, all living and working on Noongar Boodja. Our approach is guided by Indigenous knowledge systems, Indigenous standpoint theory, and Indigenous research methodologies, specifically Aboriginal Participatory Action Research (Dudgeon, Bray et al., 2020; Moreton-Robinson & Margaret, 2009; Nakata, 2007; Smith, 1999, 2021).

### Background

As leaders and cultural knowledge holders, Aboriginal^1^ Elders have played a crucial role in supporting Aboriginal social and emotional wellbeing, community self-determination, and healing, for thousands of years (Busija et al., 2020; Cox et al., 2022; Gibson et al., 2020). However, as a result of ongoing colonisation, Elders have been denied the ability to wholly fulfill these cultural roles (Gibson et al., 2020). Colonisation disrupts cultural connections, silencing and diminishing the voices of Aboriginal peoples. Violent assimilation policies disconnected many Aboriginal Elders from their families, communities, and culture (Wilson & Wilkie, 1997), resulting in profoundly harmful intergenerational impacts on health and wellbeing (Atkinson et al., 2014; Healing Foundation, 2021; Hunter & Milroy, 2006; Milroy et al., 2014). Colonisation is still occurring today, through ongoing racism, oppression, and resistance to truth-telling about Australia’s violent colonial history. A stark example of this, is the recent rejection of the Indigenous Voice to parliament in Australia, and reports of rising explicit and implicit racism during and following the referendum process (BDI, 2023; NITV, 2023).

Despite the devastating impacts of colonisation, Aboriginal Elders have supported Aboriginal health and wellbeing for generations, and have maintained strong cultural connections. Recently, Aboriginal Elders have also played an important role in guiding those who work in the health and mental health system to work in culturally safe ways (Wright et al., 2015, 2023). This is important, because many health and mental health services fail to provide culturally safe care (Milroy et al., 2022, 2024; Wright et al., 2019). Healthcare professionals often have limited understanding about cultural safety, and are unprepared and ill-equipped to work with Aboriginal peoples (McGough, 2016; McGough et al., 2018).

In this paper, we introduce the Cultural Exchange Program, where relationships and trust were developed between local Aboriginal Elders and non-Aboriginal mental health and wellbeing researchers as part of an experiential learning process. The aim of the Cultural Exchange Program was to increase the capacity of non-Aboriginal mental health and wellbeing researchers to work in culturally safe and ethical ways, as guided and determined by the Elders. Here, we examine the transformative impacts of the Cultural Exchange Program from the perspectives of the Aboriginal Elders (authors C.H., F.P., C.P., M.P., M.T.) and non-Aboriginal participants (authors K.D., J.A., E.P.C., C.D., S.K., M.P.) through APAR (Dudgeon, Bray et al., 2020).

### Moving from cultural competency to cultural safety

To address the health disparity between Aboriginal and non-Aboriginal people (Australian Institute of Health and Welfare, 2020, 2021), there has been a focus on training which develops the cultural awareness and competency of healthcare professionals (Downing et al., 2011, Downing & Kowal, 2011). However, there is mixed evidence as to whether interventions which aim to improve cultural competency have led to any meaningful improvements (Curtis et al., 2019; Drevdahl et al., 2008; Truong et al., 2014). Although an important first step, cultural competency/awareness focuses narrowly on *understanding* Aboriginal peoples and cultures (Curtis et al., 2019; Downing et al., 2011). This approach reinforces the dominant culture as the norm, and Aboriginal ways of being as “other” (Downing et al., 2011; Russell-Mundine, 2012), and also implies that competency can be achieved (see Curtis et al., 2019 for consideration of definitions of cultural competency). Further, cultural competency courses are often not aligned with Aboriginal ontologies (ways of being) and epistemologies (ways of knowing). For instance, these courses are typically developed by and for non-Aboriginal peoples, using Western teaching styles and knowledge systems.

There is now growing recognition of the role that cultural safety plays in achieving equitable health and wellbeing outcomes, above and beyond cultural competency/awareness (Australian Health Ministers’ Advisory Council, 2016; Curtis et al., 2019; Laverty et al., 2017; Merritt et al., 2018; Milroy et al., 2022, 2023, 2024; Parker & Milroy, 2014; Smith et al., 2021). Ahpra (2019) offers the following definition of cultural safety: “*Cultural safety is determined by Aboriginal and Torres Strait Islander individuals, families, and communities. Culturally safe practise is the ongoing critical reflection of health practitioner knowledge, skills, attitudes, practising behaviours and power differentials in delivering safe, accessible, and responsive healthcare free of racism*” (pg. 5). The emphasis on *critical reflection* in relation to power is important in this definition, as it aligns with literature that asserts that healthcare professionals must commit to reflective practice. Reflexivity (or self-reflexivity) involves critically challenging one’s own culture, worldview, and position of power as part of lifelong learning that addresses race privilege (Fredericks, 2008; Olmos-Vega et al., 2023; Russell-Mundine, 2012).

### The importance of reflexivity in health and mental health research

In the context of research, reflexivity allows researchers to interrogate the broader systemic ways that knowledge is created, presented, and affirmed (Nilson, 2017; Russell-Mundine, 2012), as well as how the biases and values inherent in knowledge systems can reinforce or challenge power structures (Vadeboncoeur et al., 2020). For example, this includes acknowledging that the scientific paradigm has privileged Western over Indigenous knowledge systems (Bullen et al., 2023; Dudgeon et al., 2010; Sibbel, 2004; Suchet, 2007), to the detriment of Aboriginal health and wellbeing.

The history and practice of health and mental health research has directly contributed to the colonisation of Indigenous peoples throughout the world (Dudgeon et al., 2010; Fogarty et al., 2018; Parker & Milroy, 2014; Smith, 2021). In Australia, research “on” Aboriginal and Torres Strait Islander peoples has occurred through a lens of misconception, racism, and deficit (Bullen et al., 2023; Dudgeon et al., 2014, 2023; Nakata, 2007). In 2016, the Australian Psychological Society (APS) formally acknowledged their role in conducting research that benefitted the careers of researchers over improving Aboriginal and Torres Strait Islander wellbeing, and for excluding Indigenous knowledges. The APS apology was an important moment and a catalyst for decolonial systems change (Carey et al., 2017; Dudgeon, Carey, et al., 2020; Dudgeon, Darlaston-Jones, et al., 2020). Since then, the National Health and Medical Research Council’s guidelines have been developed to support ethical research with Aboriginal and Torres Strait Islander peoples, and quality appraisal tools have been developed to assess the quality of research from Aboriginal and Torres Strait Islander perspectives (Harfield et al., 2020; Huria et al., 2019). However, ongoing reflexivity and committed action are necessary to ensure the APS apology translates to meaningful change. In particular, non-Aboriginal researchers whose work impacts Aboriginal peoples have a responsibility to engage in reflexive practice, understand the continuing impacts of colonisation, and to decolonise their standpoint and practice.

### The cultural exchange program

Despite the importance of reflexivity in the provision of culturally safe healthcare and research (Milroy et al., 2022; Smith et al., 2021, 2022, 2023), there remains limited research into approaches which would facilitate reflexive practices. The Cultural Exchange Program was developed to encourage reflexivity among non-Aboriginal mental health and wellbeing researchers, to ultimately encourage culturally safe research processes through experiential learning. The Program drew on Indigenous pedagogical practices (such as cultural immersion, yarning, storytelling and community spaces) to facilitate this process (Dawson et al., 2022; Olmos-Vega et al., 2023). Although piloted on wellbeing researchers, the Cultural Exchange Program is intended to be relevant to any person whose work impacts Aboriginal and Torres Strait Islander peoples on an individual or societal level, including clinicians, clinical providers, allied health professionals, policy developers, or undergraduate students.

The Cultural Exchange Program promoted experiential-relational learning between the Elders and participants, which occurred naturally over several months. This relational process allows for truth-witnessing through relationship building, as opposed to just “learning about” or “consuming” Australia’s colonial history from a textbook or course. Instead, truth-witnessing involves opening hearts and minds to Indigenous ways of knowing and being, with a paired responsibility to listen, remember and pass on these learnings (Nagy, 2020).

### The current study

The aim of this study is to examine the transformative experiences of the Cultural Exchange Program. These transformative experiences are considered from the perspectives of both the Aboriginal (Noongar) Elders, and non-Aboriginal participants, using qualitative data. To explore the transformative impacts, we adopted an Indigenous research paradigm that challenges Western assumptions of science, methods, and measurements (Dudgeon et al., 2010; Martin & Mirraboopa, 2003; Rigney, 2001; Smith, 2021).

## Methods

### Aboriginal Participatory Action Research (APAR)

This research follows an APAR methodology (Dudgeon, Bray et al., 2020), designed to centre Aboriginal voices and knowledges, through a strength-based approach (Dudgeon et al., 2021). Aboriginal governance and leadership were ensured through engaging Aboriginal academics, community leaders/co-researchers, and local Elders across all stages of the Cultural Exchange Program. For example, the Cultural Exchange Program was conceptualised and co-designed through a collaboration between two Aboriginal-led teams: the Transforming Indigenous Mental Health and Wellbeing (TIMHWB) team, and a local Aboriginal community-controlled organisation (Langford Aboriginal Association: LAA). An Aboriginal community co-researcher was employed to develop and facilitate the Cultural Exchange Program, and was involved as a leader throughout. In line with an APAR approach, Elders and participants were actively involved in the data analysis, through providing input into the interpretation of results. Elders and participants co-created the results and the written publications from this research, ensuring shared agreement over outputs. All Elders and participants are authors on this paper.

### Research Governance

The research was undertaken at the joint request of LAA and the TIMHWB Aboriginal team leaders. The research responded to a priority determined by community: to build relationships between Aboriginal Elders and non-Aboriginal mental health and wellness researchers, in order to support culturally safe research processes. The Aboriginal team leaders provided senior Indigenous governance over the project and decision making in the project, including how the Cultural Exchange Program should be run, the focus of the research question, the research process, and translation of results. The three Aboriginal team leaders have joint governance over the research outputs.

This research is aligned to the NHMRC Guidelines for Ethical Research with Aboriginal peoples and was approved by the Aboriginal Health Council of Western Australia (HREC1057). Written informed consent was obtained from the Elders and participants.

### Participants

#### The Elders

The Cultural Exchange Program was developed and delivered on Whadjuk Noongar Boodja, Perth, Western Australia. Wadjuk is one of 14 Noongar groups in this region. Honouring this location, five Noongar Elders were invited to participate in the program (3 female, 2 male). The Cultural Exchange Program occurred as a two-way exchange of communication, although the exchange of culture was intended to occur primarily in one way (i.e., knowledge exchange from Noongar Elders to non-Aboriginal researchers). Elders were invited to take part in the program by LAA based on demonstrated community leadership, and involvement with programs at LAA. Elders were reimbursed for their time and sharing of knowledges.

##### The non-Aboriginal participants

Participants (*n* = 6; 25–52 years; 5 female; 1 male) were non-Aboriginal mental health and wellbeing researchers whose work impacted Aboriginal peoples. The participants had been working in a full-time capacity with Aboriginal and Torres Strait Islander peoples for between two weeks and two years. The researchers had a basic level of cultural awareness and responsiveness, as determined by the Aboriginal leadership team, which was important to ensuring the safety of the Elders.

### Procedure

The Cultural Exchange Program was held in-person at LAA. Noongar Elders and non-Aboriginal participants met over eight workshops across 3 months. Aboriginal pedagogies were used as a method to facilitate relationship building, trust and learner reflexivity (Dawson et al., 2022). For example, during the workshops, the group yarned together, shared stories, engaged in art (e.g., drawings) and shared meals together. This provided a personal way of engaging with Aboriginal ways of knowing, being, and doing. The chosen location was determined by the Elders as it was a safe and familiar space for them. This space allowed the participants to explore the community garden, appreciate LAA member’s art, and enabled the employment of an Aboriginal caterer. Spacing the workshops across several weeks facilitated reflexivity through encouraging participants to reflect on their own biases present in their everyday lives, and also facilitated relationship building over time.

Each workshop was facilitated by an experienced Wilman/Goreng Noongar woman who had long term relationships with the Elders and LAA. Each workshop lasted approximately four hours and focused on a different topic. Although the facilitator had a set protocol for the workshops, the conversations were also allowed to flow naturally as led by the Elders (see Appendix 1 for summary of topics). This structure allowed sharing of lived experiences, including of racism, Stolen Generation experiences, and connection to Country, kinship, culture, and spirituality.

Each session commenced with a Welcome to Country by an Elder or an Acknowledgement of Country by a participant, followed by introductions. Introductions involved sharing one’s name, connection to ancestry and Country (where relevant), where people live using the local Aboriginal name, and a check in word. The purpose of these introductions was to ground the participants, and connect them to each other in a rigorous and culturally safe way accustomed to yarning (Bessarab & Ng’andu, 2010).

After completing the Cultural Exchange Program, the participants attended a graduation ceremony with the Elders at LAA, as part of a community breakfast event. Prior to the commencement of the Program, the participants met with the facilitator to receive pre-program training, to help facilitate a culturally safe environment for the Elders. For example, participants learnt about appropriate language, culturally informed ways of introducing oneself, and were encouraged to examine their racial biases and how these impacted assumptions in decision-making.

### Measures

This research adopted data collection methods and measures which facilitated further reflexive thinking in the researchers (Olmos-Vega et al., 2023). There were two sources of data collected and analysed: 1) qualitative data from semi-structured interviews/Focus groups, and 2) qualitative data from the Stories of Most Significant Change.

#### Semi structured interviews/Focus groups

Approximately one month after completing the Cultural Exchange Program (before the graduation), the Elders and participants gathered separately to reflect on the transformative impacts of the Cultural Exchange Program. Both groups answered a set of questions as part of a semi-structured yarning interview (see Appendix 2 for the questions). For example, participants were asked questions relating to their motivation for taking part in the Cultural Exchange Program, reflections of themselves before the program, and benefits of the program. Elders were also asked about whether they felt their knowledge and lived experiences were valued during the program. These questions were designed by the Aboriginal leadership team.

The Cultural Exchange Program facilitator used a yarning method to go through the questions with the Elders in a group, taking detailed notes and transcriptions of the conversation. Yarning is a way that Aboriginal peoples refer to sharing knowledge that is reliant upon relationships and cultural protocols (Dean, 2010), and can also be used as a powerful research tool for collecting data (Bessarab & Ng’andu, 2010; Kennedy et al., 2022). Yarning is a culturally grounded relational pedagogy conductive to APAR, as it empowers participant voice and positions participants as knowledge holders and research contributors. The facilitator used social yarning to help create a safe space, starting with each member introducing themselves and connection to Country. Research topic yarning was then adopted to address the research questions (Bessarab & Ng’andu, 2010). This also occurred as a collaborative yarn, as the group openly discussed the impacts of the Cultural Exchange Program, reflecting on shared learnings and experiences. This session was hosted at LAA.

The non-Aboriginal participants met as a group to collaboratively discuss the questions at the University of Western Australia. The group started with an Acknowledgement of Country, a check in word, a minute of silence, and ground rules. The group collaboratively reflected on the impacts of the Cultural Exchange Program, in a process akin to collaborative yarning (Bessarab & Ng’andu, 2010). One participant took summary notes of the conversation, projected on a screen to ensure group validation.

#### Stories of Most Significant Change (SMSC)

In their own time, the Elders and participants completed an individual reflexive exercise whereby they wrote about their experience participating in the Cultural Exchange Program. The Stories of Most Significant Change extraction technique (Davies & Dart, 2005) is valuable for assessing programs which strive for social change and learning journeys as it directly encourages a self-reflection of these changes. Here, we used an adapted version of this technique, which has been used previously in APAR and considered a culturally safe qualitative approach for investigating mechanisms of change (Dudgeon et al., 2022).

### Thematic analysis

**Figure 1.**
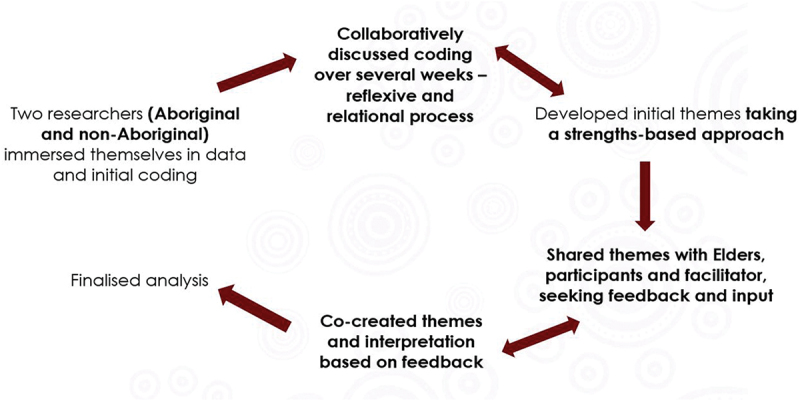
Aboriginal participatory action research approach used through a reflexive thematic analysis. Bold font highlights the steps which were primarily influenced by an Aboriginal participatory action approach. Sometimes each step was aligned with both approaches.

The analytical approach combined APAR (Dudgeon, Bray et al., 2020) with reflexive thematic analysis (Braun & Clarke, 2021), as outlined in Figure 1. For the constructs being unpacked here, reflexive qualitative research methods were most appropriate (Braun & Clarke, 2013), as they allowed for Aboriginal voices to be empowered and an understanding of inner transformation. A reflexive thematic analysis (Braun & Clarke, 2021) was conducted to determine the transformative experiences of the Cultural Exchange Program, from both the Elders’ and participants’ perspectives. In line with an inductive approach, the coding of data was open. The research question itself also evolved through the coding process. That is, under Aboriginal governance, the coders refined the research question after immersing themselves in the data. Through this process, the decision was made to focus on the following question: *What were the transformative experiences of the Cultural Exchange Program for both the non-Aboriginal participants and Aboriginal Elders?*

Two independent coders who did not participate in the Cultural Exchange Program (one Aboriginal: B.S., and one non-Aboriginal: J.C.) worked collaboratively on the reflexive thematic analysis, in the spirit of two-eyed seeing (Bartlett et al., 2012). The focus group and SMSC data were analysed together, separately for the Elders and participants. The coders followed a recursive process for thematic analysis (Braun & Clarke, 2006), as per Figure 1. First, both coders immersed themselves in the data. One coder then generated initial codes, focusing specifically on transformative experiences. Through an iterative approach, the second coder then reviewed these initial codes and generated their own codes. The two coders engaged in a collaborative and reflexive process, unpacking the meaning of the data, and discussing any discrepancies in coding through a relational process which lasted several weeks. In line with APAR, the coders adopted a strengths-based coding approach, and when differences in coding occurred, the Aboriginal perspective was prioritised. The codes were then combined to form broad overarching themes, separately for the Elders and participants.

In line with an APAR approach, feedback and input was then sought from the 1) Elders, 2) program facilitator and 3) participants, regarding the initial themes. The purpose of this step was to verify the accuracy of interpretation, as well as to co-create the themes. For example, this feedback process allowed for changes to be made to the language used, clarification of quotes, and framing of themes. The feedback session with the Elders was led by the Aboriginal program facilitator, supported by one of the researchers involved in the data analysis (JC). It was the Elders’ choice to collectively read the results, taking it in turn to read the results out loud. This process reinforced a sense of collective ownership over the data, and empowered the voices of the Elders. The session occurred at LAA, over a shared meal. The Elders were financially reimbursed.

The participant feedback session occurred as an online/in-person hybrid. The results were shared on screen, and the group collectively discussed and provided feedback. Following this session, written feedback was also collected. This process further supported reflexive thinking, encouraging participants to reflect on how the Cultural Exchange Program had continued to facilitate change beyond the life of the Program.

Through the above process, three key themes were identified with the Elders and four key themes with the participants. As a way of privileging Elder voices, Elder results are reported first.

## Results

### Themes for Elders

#### A desire to educate others to bring about change

The Elders expressed a desire to take part in the Cultural Exchange Program in order to provide non-Aboriginal people an insight into their lives, and to share knowledge regarding how Aboriginal people lived prior to invasion, and during ongoing colonisation. The Elders recognised the ignorance of many non-Aboriginal people regarding Australia’s racist history, sharing that “*some of them have no clue of how we lived, [the] struggles we went through*”. The Elders expressed a readiness to stand strong together, with the intent of sharing personal stories and educating others about the truth.

Elders were able to recognise the significant value of lived experience and cultural knowledges, and expressed a desire to educate the participants about these. The Elders expressed feeling “solid” in themselves as teachers, and as experts of personal lived experiences.

We all have stories, we are sharing our stories. Staying strong, standing together.

We [are] ready to tell the truth to these young people … Tell them about our life … how we survived, how we struggled, about Stolen Gen[eration], how we were taken away, how we got back to our family, let them know our life.

The Elders shared a desire to work together with non-Aboriginal people to bring about change. As part of this process, Elders saw the importance of educating non-Aboriginal people so that they can also share these learnings and bring about system change. The Elders recognised that Aboriginal peoples represent a minority of the population, identifying the importance of non-Aboriginal people who are “*on our side and that will try and bat for us*” (quote from feedback session with Elders). In particular, the Elders saw the participants as having an obligation to amplify Elder’s voices.

We all wanted to share, to tell, we are the experts in our life, we wanted to share, we need people who are not Aboriginal who will have high positions [to hear], we need them to change the system … have to educate non-Aboriginal people who are sympathetic and listen to us to change the system for everybody.

We want non-Aboriginal [people] to know our stories.

#### Truth telling – healing and empowering

A salient theme for the Elders was the power of truth-telling. Through truth-telling and sharing lived experiences as part of the Cultural Exchange Program, the Elders experienced a journey of growth and healing. Elders shared these experiences with pride and felt liberated in exposing the truth about the historical and contemporary racism entrenched in Australia.

Coming from all of us, we wanted to let it all out, we weren’t holding back once we got started, they were shocked to hear what was coming, was healing letting it all out, all talking not shame, not have things hidden anymore.

Although the Elders’ lived experiences included difficult personal experiences, sharing these stories reinforced a sense of cultural pride, strength, resiliency, and empowerment. Sharing stories and lived experiences reinstated Elders’ sense of hope and optimism for the future.

I feel happy and excited when telling my story about my life, my days of being taken and put in [a] Mission. I love talking about my culture and language and the struggles my family and ancestors went through.

Telling our stories of the past and the lived experiences for me helps in a healing process. Has made me feel proud of the resilience and strength of my culture.

Make me a stronger person, brought everything out from when you were young, each time I came back I felt stronger.

For some Elders, this was the first time that they had ever shared lived experiences with non-Aboriginal people. Building relationships with the non-Aboriginal participants, who bore witnesses to the truth, helped some Elders heal painful emotions.

I have become more tolerant towards non-Aboriginal people. Continuing to let go of anger and bitterness towards the colonisers. To be a better man.

#### Cultural respect and two-way learning

The Elders reported that the participants engaged in authentic truth witnessing, and demonstrated cultural respect. In doing so, the Elders felt acknowledged, validated, seen, and heard. For example, the Elders picked up on the group’s willingness to learn over time, and could tell that the knowledges which they were sharing were valued by participants.

They picked up on our names, they must have like[d] what we had to say, they were there waiting, didn’t want to miss anything, they were very engaged in the conversation.

[It] worked as they wanted to come back and learn more, they were engaged in our stories …

I think it has made a difference cause we told our stories, they heard from each of us, continue to acknowledge what we spoke about … they acknowledge us.

Elders expressed a desire to learn from the participants, as part of a two-way learning process. For example, the Elders learnt about the participants’ culture, and built relationships with the participants through this process. A desire to learn about the varied cultures of the participants was an unexpected outcome of the program.

To me it was really [great] to understand other people’s cultures. They were in some ways similar to mine, which I would have never expected.

### Themes for non-Aboriginal participants

#### Respect for Elders through truth witnessing and relationship building

A key theme for participants was a deep respect for Elders and their knowledges, which developed through authentic truth-witnessing and relationship building. Participants learnt about the lived experiences of Aboriginality and developed authentic relationships with and respect for the Elders. Participants were inspired by the way the Elders openly shared their lived experiences and cultural knowledges with kindness, generosity, and trust. Despite the hard experiences faced by the Elders, the Elders graciously shared personal past stories without resentment.

To be inspired by the Elders and the way they always interacted with us with optimism, and an open heart, and responded to our ignorance with kindness and grace and generosity instead of anger or resentment or fear.

I am blown away by the grace with which [the] Elders have faced difficult experiences and the generosity that allows them to come to us questioningly but still willing to teach us, and trust us to do the right thing with the knowledge they have shared. The weight of this responsibility is real…

Participants saw the privilege in being able to share this time with Elders, and develop genuine connections with the Elders. However, they described this experience as “bittersweet”, as participants also witnessed the truth about Australia’s racist and oppressive history, the impacts of colonisation, and contemporary experiences of racism. This experiential learning provided a more intimate and nuanced understanding and appreciation of Australia’s history than participants had gained through reading about these events, which in turn triggered emotional responses.

There in the stories, we were faced with the reality of the alienating and unrelenting racism the Elders had faced throughout their entire lives, from the day they were born … Hearing personal experiences made me feel sad, empathic, and ill from how awful the experiences were.

Despite what the Elders have experienced in their lives (e.g. as they described, “being treated badly by foreigners in their own country”), they still welcomed us, opened- up to us, taught us, and trusted us. Their trust and friendship is such a privilege, it helped me understand how important truth telling is…

#### Unsettling colonial realities

Participants began to realise and name their ignorance of Aboriginal lived experiences. The experience facilitated an awakening to issues they had not previously thought about in their lives. Participants were faced with the unsettling reality of their settler-colonial identities, specifically, the entrenched racism in contemporary Australia.

Racism had never really impacted [us] before, [there was] awareness, but not active participation in or thinking about or advocacy of [racism], so [we] didn’t think about [racism] a lot. (collective group reflection)

[The] majority of my life the issues faced by Aboriginal and Torres Strait Islander people did not factor into my life at all and it was something that I didn’t take much notice of.

Truth-witnessing facilitated continuous learning and understanding of Aboriginal historical and contemporary lived experiences. This ranged from being more attuned to racist language used towards Aboriginal and Torres Strait Islander peoples, to becoming more aware of the ongoing consequences of colonisation and intergenerational trauma.

The most significant change that I have seen in myself is in regards to my appreciation of the day-to-day struggles that Aboriginal peoples have experienced in direct consequences of colonisation, the Stolen Generation and the overt and systemic racism that came from it…. I did not know how many Aboriginal people had to choose between reunification with their children and a complete [severance] of their Aboriginal ties…

#### Learning to reflect on ways of knowing, being, and doing

The Cultural Exchange Program encouraged an inwards-looking, transformative learning journey, that promoted reflexivity and epistemic vulnerability. That is, participants were open to altering and adjusting their ideas and beliefs.

Slow down, be mindful and thoughtful, think about what we are doing here … Breaking away from traditional ideas of psychology, acknowledging standpoint and privilege, truth-telling vs the rigidity of science. (Collective group reflection)

Throughout the [Cultural Exchange Program] I have struggled to comprehend how the cultural exchange process will actually impact my way of working with Aboriginal peoples and who I am as a researcher … But I understand that this is me learning to “walk in two worlds” and consider other ways of knowing, being, [and] doing.

Participants also acknowledged and critically reflected on their own biases and values, and where these stemmed from.

I used to be one of those people that couldn’t understand why Aboriginal people hadn’t moved on from the trauma of their past, now I don’t understand how I could have ever expected that in the first place.

Before the Cultural Exchange Program, I did not feel well-equipped to know how to manage situations when people around me made a racist comment or remark. I sometimes stayed silent, but showed a disgruntled face or said “that’s not right” because I disagreed. I think this was because I did not know what to say … Through the learnings and understandings from the Elders, and the personal connections formed with the Elders, I have learned that I may never fully have the “right” thing to say, but to not say anything is to remain complicit.

Sometimes this reflection was difficult. Participants sat with the discomfort of identifying as settlers, questioning their roles and responsibilities as non-Aboriginal researchers, and recognising their position of power and privilege. Participants were grateful for these transformational learnings, whilst acknowledging that this learning would be continuous.

The guilt felt from my ancestors who profited in Australia at the expense of the First Nations peoples. [is real]

… that too can be perceived as exploitation and ultimately I need to be accountable for the ways I behave and conduct research if I want to earn my right to work in this space. To be able to receive this kind of feedback with humility and openness is really key, because at the end of the day, we don’t see our privilege.

I can’t expect myself to undo a lifetime worth of conditioning in a few weeks but I feel at least I am on a path of awareness and I am better able to acknowledge my own privilege.

When reflecting on these themes with the participants as part of the feedback loop, participants highlighted how the Cultural Exchange Program had been a “trigger point” in their lives for many conversations. The Program encouraged participants to engage in ongoing reflexivity of their own biases even after the program ended, as a continuing learning journey.

#### A call to apply learnings to action social justice

Participants reported a commitment to social justice and action after completing the Cultural Exchange Program. For example, participants expressed a desire and commitment to ensure that academic knowledge and practice were not used as tools of colonisation. This also extended to a commitment to social justice in participants’ everyday lives.

As non-[Aboriginal] researchers, it can be very easy to choose to not speak up when faced with racism or malpractice as it is “too hard” or “not my problem”, but now I feel that we owe this to the Elders for the stories shared to speak up when no one else does, and ensure that we use that knowledge for good … and [to make] the effort to engage with Aboriginal issues not just in our work, but outside our work in our friendships and personal lives so we understand lived experience and not just the academic interpretation of current issues.

I have also become more aware of respecting and acknowledging Aboriginal culture in my everyday life, this includes making sure my work team say an Acknowledgement of Country before every team meeting, supporting local Aboriginal run businesses…. I do I feel more inspired to be an advocate even if it is just in these small ways.

Participants reported a commitment to “speak up” when they identify social inequities and racism, even when this was challenging or discomforting to do so. Sometimes, this commitment was perceived as a responsibility, to pay forward what they had learnt from the Elders and to demonstrate their commitment to social justice.

My biggest learning and significant change has been to speak up. It is everyone’s responsibility to make change and speak up: change starts with each of us.

Knowing how to have those very tricky conversations is really important too. I have definitely felt more confident and responsible to speak up, but sometimes it goes well, and other times it does not.

## Discussion

This paper explores the transformative impacts of an experiential learning opportunity between local Aboriginal Elders and non-Aboriginal participants (mental health and wellbeing researchers). For the Elders, the Cultural Exchange Program provided an avenue for educating non-Aboriginal people about Aboriginal culture, the impacts of colonisation, and lived experiences of Aboriginality. Through sharing lived experiences and truth-telling, the Elders also experienced growth and healing, and felt Aboriginal culture was respected. These finding aligns with previous healing experiences for Elders following yarning and storytelling (Gibson et al., 2020), and demonstrate the healing power of truth-telling. The Elders were also motivated to learn about the non-Aboriginal participants’ culture as part of two-way learning and relationship building.

In the words of Kanien kehaka (Mohawk) scholar, Taiaiake Alfred, decolonisation involves “*embracing the discomfort of the unsettled existence of an ally committed to the strength and well-being of Indigenous nations”* (Alfred, 2018). Through the Cultural Exchange Program, the non-Aboriginal participants developed deep respect for the Elders, were challenged to face unsettling colonial realities, and were encouraged to reflect on and challenge their own world beliefs, and to turn this into action in the fight for social justice. At times this experience was difficult, and participants questioned their position as non-Aboriginal researchers working in this space.

The Cultural Exchange Program encouraged the participants to challenge their own culture and ways of thinking, as part of lifelong commitment to reflexivity and decolonisation. Instead of asking *what information do I need to learn about*, reflexivity asks, *who am I and what is my responsibility in response to this information* (Smith et al., 2022). Through developing authentic relationships with the Elders, the non-Aboriginal participants critically reflected on their roles and responsibilities as non-Aboriginal mental health researchers. It is also likely that reflexivity would result in better mental health practitioners, through supporting them to work in more culturally safe ways with people from all cultures. These results build on evidence which highlights the centrality of reflexivity as an avenue for developing cultural respect and promoting cultural safety (see Peter Smith et al., 2021, 2022).

In order to ensure that Aboriginal and Torres Strait Islander peoples live healthy, thriving lives, health services must be culturally safe. This starts with ensuring that healthcare workers are equipped to work with Aboriginal and Torres Strait Islander peoples, above and beyond “achieving” cultural competence or awareness. This learning cannot be achieved in a one-off cultural awareness/competency course. Similarly, cultural immersion programs must be long enough to allow for ongoing reflection and genuine relationship building, rather than only lasting a few short days and providing “a quick dip into another culture” (Smith et al., 2015). The Cultural Exchange Program ran over several weeks to allow for longer reflection time and relationship building. The relationships developed were guided by authenticity, kindness, and family in a kinship sense. Kinship relationships involve obligation and reciprocity: not because one has to, but because one wants to.

The Cultural Exchange Program was offered to a group of non-Aboriginal mental health researchers, although this program is likely to benefit a much broader audience, including health care providers, educators, and university students. Indeed, other programs run with Aboriginal Elders have also shown transformative impacts (Wright et al., 2015, 2023). Looking ahead, future research could consider how the Cultural Exchange Program might be implemented in other regions, with local Elder groups. It is important to note that the outcomes of the Cultural Exchange Program were the result of the specific group of Elders and participants, and therefore, it may not be possible to re-create these same outcomes with different participant groups. This is not a limitation of the current program, but rather, aligns with a place-based approach. In line with such an approach, the outcomes would be relevant to the specific location or community where the program was run, and the rigour of the relationship building, co-design, and reflexivity processes throughout the program.

To ensure that decolonising endeavours do not unintentionally perpetuate acts of colonisation (Opara, 2021), the *process* of these endeavours must be carefully considered (Lipscombe et al., 2021; Milroy et al., 2022). Although the Cultural Exchange Program is intended as a map that other Aboriginal and Torres Strait Islander community groups can adapt to meet local community needs, the process of any adaptation should be carefully considered. There are several principles which should guide the development of similar cultural exchange programs. These guiding principles include:

- *Aboriginal led/governed*: Aboriginal and Torres Strait Islander peoples should be engaged with all stages of program development, including from conceptualisation to implementation and any evaluation. The program should be led by an Aboriginal community-controlled organisation and co-designed with local Elders, Aboriginal leaders or knowledge holders who have connections to the Country that the program is delivered on. This will also facilitate a place-based approach to the Program.
- *Community relationship building*: The program should allow for relationship building between local Elders and the participants over an extended period of time. There should be pre-existing relationships and community connections between the organisations of the Elders and the participants, likely established through the program facilitator. The facilitator also plays a crucial role in creating a safe environment for the Elders and participants.
- *Learning is grounded in Aboriginal ways of knowing, being, doing*: The program should follow Aboriginal knowledge systems and pedagogies (Martin & Mirraboopa, 2003) such as adopting culturally informed teaching and learning styles, and local cultural protocols.
- *Reflexivity and readiness for truth-witnessing*: Reflexivity must be a core feature of the experiential learning process (so that we are not just learning about the “other”). Participants must be selected based on their ability to act respectfully, and recognise the privilege and honour of spending time with Elders.

## Conclusion

In the Cultural Exchange Program, a culturally safe space was created through genuine co-design, community engagement and empowerment, and culturally guided methods of knowledge sharing. In this environment, Aboriginal Elders and non-Aboriginal participants engaged in independent but intertwined transformative journeys, built on the foundation of friendship, trust, and reciprocity.

## Supplementary Material

Appendix 1: Schedule

Appendix 2

## Data Availability

In line with the Ethics approval for this project, data is not publicly available for this project

## Note

1. We respectfully use the word Aboriginal at the request of the Aboriginal Elders who were part of this project, and given the local context of this research on Noongar Country. We use the word Indigenous when referring to Indigenous peoples globally, or knowledge systems.
