# Supplementary material for: Truth telling and truth witnessing: results from a transformative experiential learning program between Aboriginal Elders and non-Aboriginal researchers
Source: Aust J Psychol. 2024 Nov 14;76(1):2425624. doi: 10.1080/00049530.2024.2425624 (PMC12218524; doi:10.1080/00049530.2024.2425624)
Supplement: Appendix 1: Schedule [file RAUP_A_2425624_SM7900.pdf]

Weekly Schedule:

Session 1: Introductions & families & culture

Yarn Up cards, Karda colouring in,

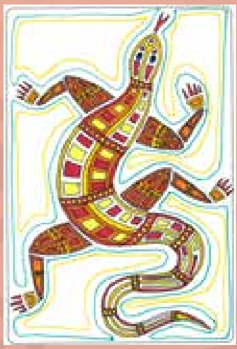
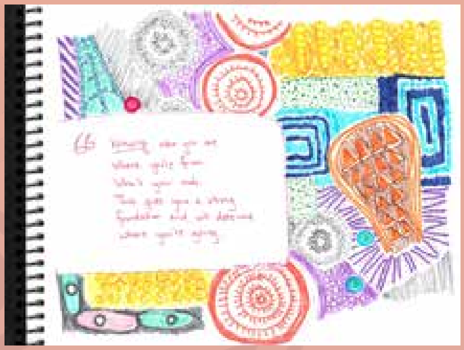
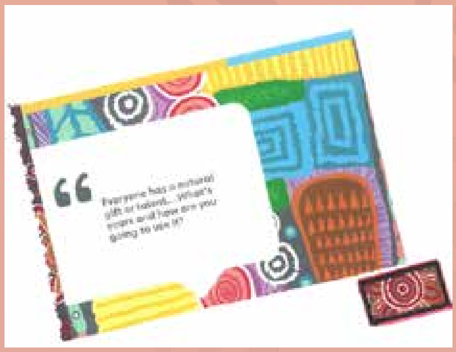
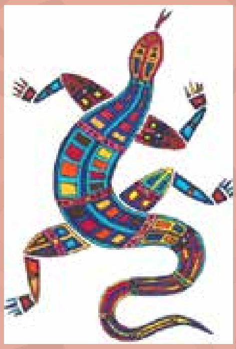

Session 2: History and childhood experiences

Question cards, Yonga colouring in

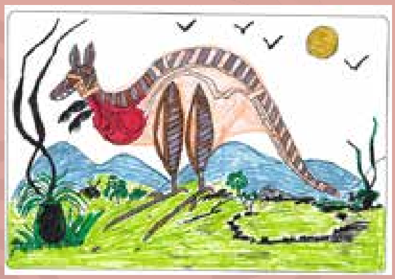
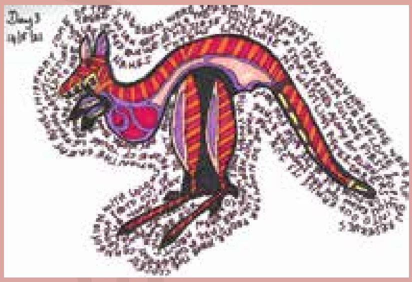
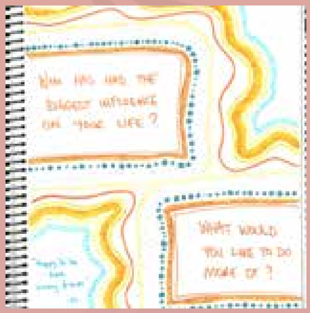
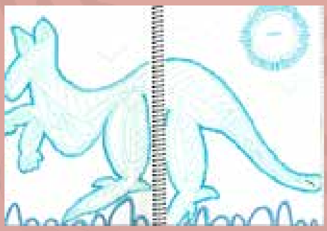

Session 3: Experiences of racism and injustice

Flower cards (life academy.com.au living essences.com.au), Australia colouring in

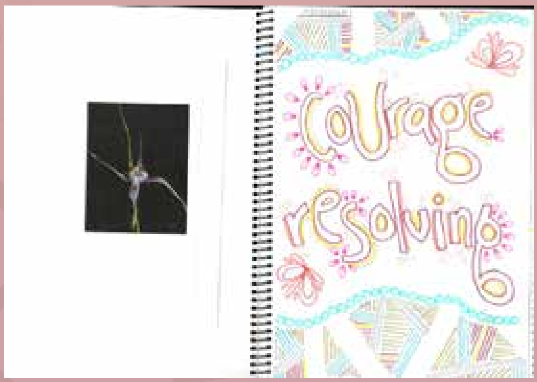

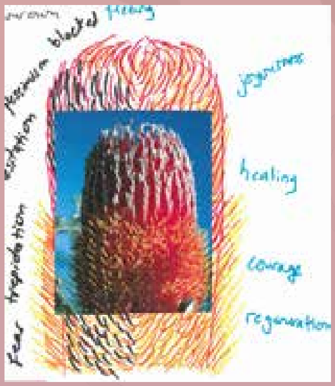

Session 4: Reconciliation, sorry, and what this means

Aboriginal picture cards, turtle colouring in

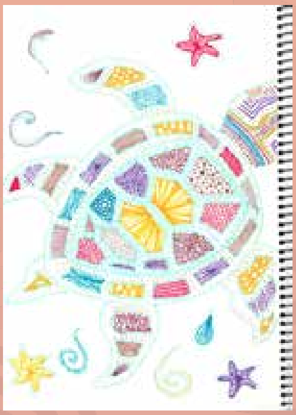

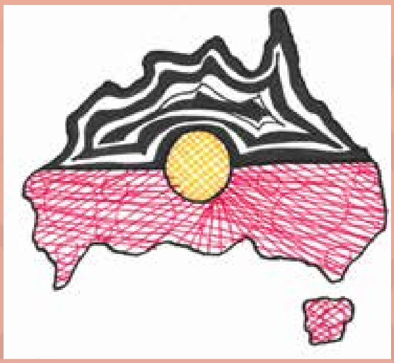

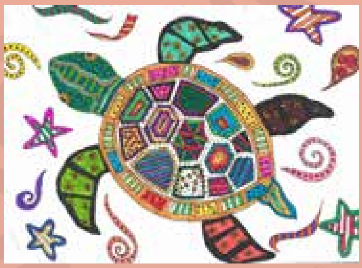

Session 5: Healing and spirituality

Aboriginal picture cards,
Reconciliation colouring in

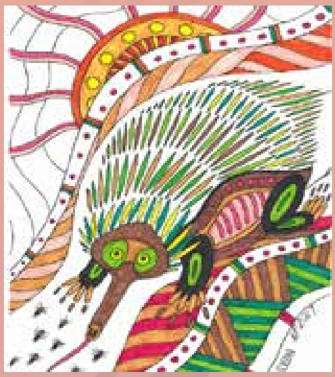

Session 6: Thinking about future and youth

Not Salmon quote cards

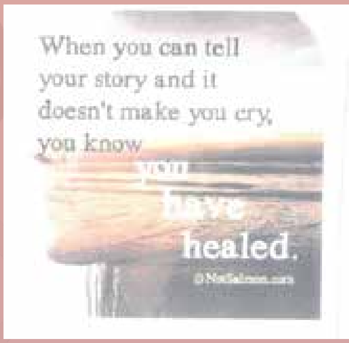

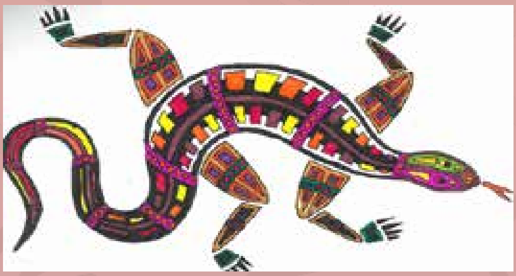

Session 7:

Magnet words, singing activity
