# Supplementary material for: Truth telling and truth witnessing: results from a transformative experiential learning program between Aboriginal Elders and non-Aboriginal researchers
Source: Aust J Psychol. 2024 Nov 14;76(1):2425624. doi: 10.1080/00049530.2024.2425624 (PMC12218524; doi:10.1080/00049530.2024.2425624)
Supplement: Appendix 2 [file RAUP_A_2425624_SM7899.pdf]

Interview Guide
Cultural Exchange Program for Researchers

Interview guide by theme

Theme	Questions/prompts for researchers (*Elders*)
Cultural humility	Tell us a bit about yourself before the cultural exchange program?<br>(prompt, specifically in terms of cultural responsiveness*, cultural humility*, and self-reflexivity*)<br>(*prompt, your impression and interaction of non-Aboriginal workforce/researchers*)<br>*defined for interviewer but not to lead the interviewee
Benefits	What benefits (wellbeing and education) have you got from the program?<br>*education/personal development
Challenges<br><br><br>Risks	What challenges did you face during the program?<br>Did anything inhibit your ability to learn or to change?<br><br>If anyone was to take on this concept of cultural awareness training, what are the potential risks for implementation for participants, facilitator, and Elders?
Mechanisms of change	What mechanisms of change were there for getting from where you were, to where you are now?
Uniqueness	Have you done any cultural awareness training?<br>If yes, how does this cultural exchange program differ from other 'cultural awareness' training that you have done?
Duration<br><br>Content	How many sessions do you think is enough?<br><br>What are the important topics to cover?<br>What is your understanding of Aboriginal research?<br>* in addition to Stories of Most Significant Change
Others	Is there anything else you would like to add about the cultural exchange program or your experience over the last few months?
